# Light people: Nobel Laureate Prof. Eric Betzig

**DOI:** 10.1038/s41377-023-01205-3

**Published:** 2023-08-08

**Authors:** Hui Wang, Cun Yu

**Affiliations:** Changchun Institute of Optics, Fine Mechanics and Physics, Chinese Academy of Sciences, 130033 Changchun, China

**Keywords:** Optical spectroscopy, Imaging and sensing

## Abstract

Super-resolution microscopy illuminated the hazy molecular world like a beacon, bringing significant changes to the fields of biology, optics and chemistry. Eric Betzig, who shared the 2014 Nobel Prize of Chemistry with Stefan Hell and William Moerner, is one of the inventors of this fantastic scientific tool. Working originally on near field optics, Dr. Betzig made a series of stunning achievements at Bell Labs before turning 35. Yet he quit, seemingly at the top of his game, to become a hands-on dad and joined the machinery business. Eventually, he missed science so much that he made a comeback and wowed everyone instantly with his work. He defies the conventional definition of “persistence” by demonstrating an alternative route to success. Plain-speaking and earnest, Dr. Betzig is sometimes honest to almost a fault, and always believes in doing practical things. He puts his heart and soul into everything he does, because he says he wants to truly live rather than simply pass through life. In science work, he is meticulous, yet he embraces risks and believes they can bring the best out of oneself. In life, he is a loving father who is always ready to take time from his busy research work to take his children to school or tennis lessons, and a caring teacher who never forgets to command the hard work of his postdocs. Please join us for a glimpse of the Nobel laureate Eric Betzig and his extraordinary life.

## Abstract (web summary)

Eric Betzig, winner of the 2014 Nobel Prize of Chemistry, believes scientists should take risks and think out of the box in order to achieve breakthroughs.

## Graphical abstract

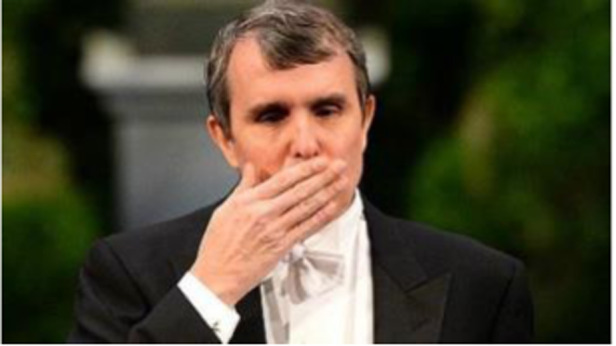

**Figure:**
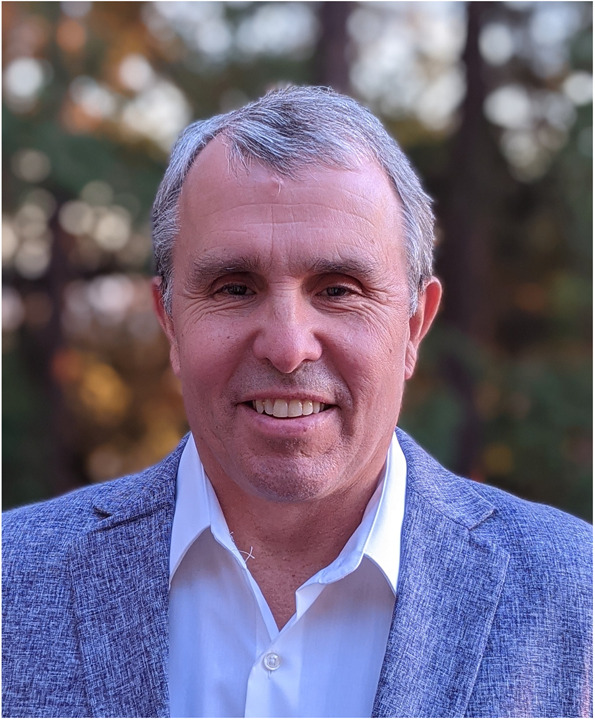

Eric Betzig is a Professor of Molecular and Cell Biology and the Eugene D. Commins Presidential Chair in Experimental Physics at the University of California, Berkeley. He also serves as Senior Fellow at the Janelia Research Campus of the Howard Hughes Medical Institute. His Ph.D. thesis at Cornell University and subsequent work at AT&T Bell Labs involved with the development of near-field optics, an early form of super-resolution microscopy. He left academia in 1995 to work in the machine tool industry, but returned 10 years later when he and his friend, Harald Hess, built the first super-resolution single molecule localization microscope in Harald’s living room. For this work, he is a co-recipient of the 2014 Nobel Prize in Chemistry. Today, he continues to develop new imaging tools to aid biological discovery, including correlative super-resolution fluorescence and electron microscopy, 4D dynamic imaging of living systems with non-diffracting light sheets, and adaptive optical microscopy to recover optimal imaging performance deep within aberrating multicellular specimens.

**1. You won the Nobel Prize in 2014 for “the development of super-resolved fluorescence microscopy”, could you tell us about how you made this important technical breakthrough?**

Prof. Betzig: My work in super resolution started back in 1982 when I went to graduate school at Cornell. We had a couple of professors working on the theory that they could pass light through a hole smaller than the wavelength of light in a black screen, which would work like a nano flashlight. This is what is now known as near field scanning optical microscopy. I worked on that in graduate school, and also at Bell Labs after I joined it in 1988, and I had a number of successes. In 1989, William. E. Moerner who shared the Nobel Prize with me later did an experiment using absorption spectroscopy to see the signature of a single molecule spectrally at near absolute zero, a few degrees Kelvin, in a crystal. So researchers started wondering if the same could be achieved at room temperature. With my near-field technique it was very easy to look at fluorescent molecules to a fraction of the wavelength of light, and I published a paper on that in *Science*. I began to think about single molecules. And a few years going on as my technique became more popular in the field, I also learned that this method of super resolution had a major limitation: the light that came out of that little hole would spread super rapidly with distance, so you had to have the hole about 20 nanometers or closer to the specimen. That was a major problem for any sample that wasn’t completely flat on a nano-metric scale. Since I was interested in ultimately trying to make an optical microscope that could look at living cells with the resolution of an electron microscope, and living cells aren’t flat at the 20 nanometer level, I grew frustrated.

**Figure:**
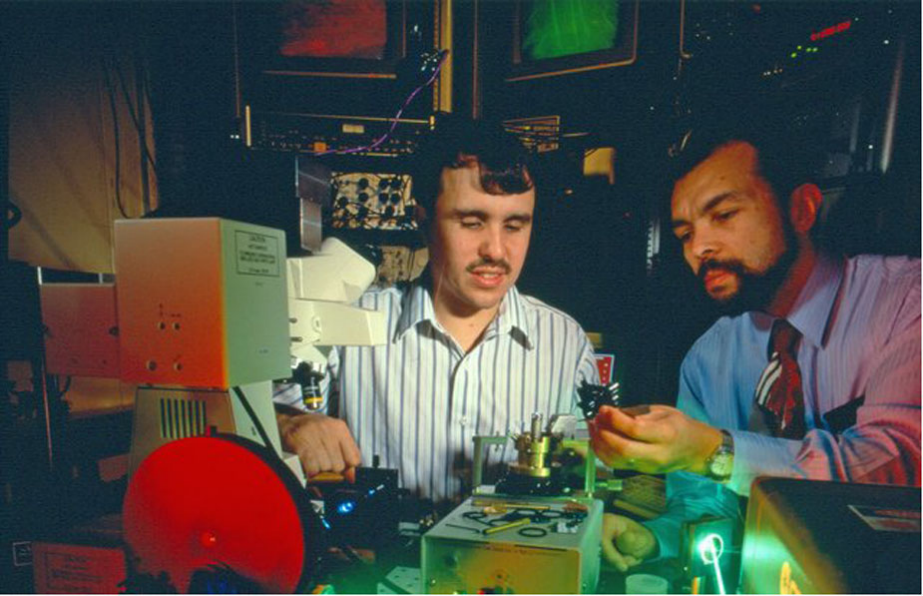
Eric and collaborator Rob Chichester in 1993 with his near field microscope at Bell Labs

**Figure:**
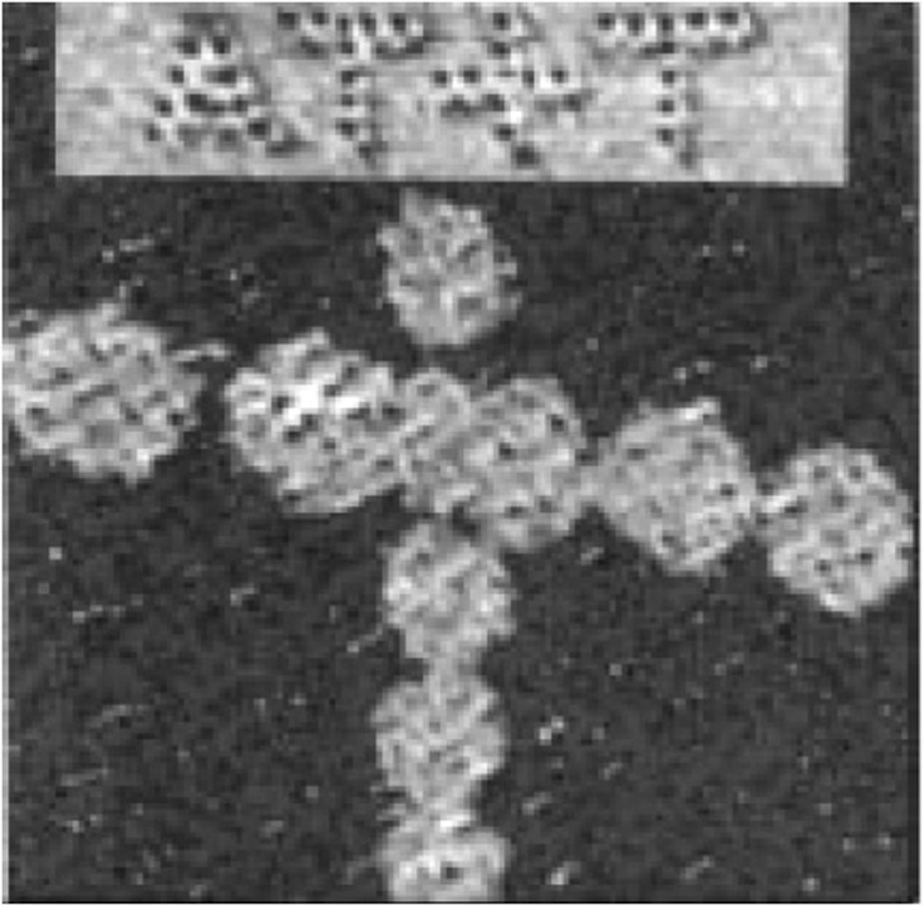
Near field optical image of magnetic recording bits Top: high density bits (black) written with super-resolution near field microscopy Bottom: low-density bits (white) written with conventional diffraction-limited microscopy

I also grew frustrated because of a trend I see in science. I think that in general academic science is mainly sequestered in bubbles and people find bubbles of like-minded people. And everybody is happy in this little bubble, everybody acting alike and everybody getting along. But it leads to mediocrity. It doesn’t lead to really radical ideas and people pushing in new directions. And furthermore, those bubbles are very much generally limited in their horizon. They’re focused on their problem. But I find that the breakthroughs in science happen when people interact at the interface between very different disciplines that don’t necessarily have a clear connection. To this day, anything that I’ve succeeded within sciences is by getting out of my comfort zone and learning more about some field that has nothing to do with optics, and how optics can be applied to it.

In addition, as near-field microscopy became a hot field, a lot of people who had no experience and no knowledge of the limitations jumped in and created so much noise that the signal of good work was drowned out. Eventually I was so frustrated that I quit Bell Labs and just basically stayed at home and helped to raise our first baby. Then I had an idea one day that if I had single molecules all glowing in different colors, then I could find their center just by fitting the point spread function and make a super resolution map of their locations which would mean a super resolution image in the far-field. Of course at the time I couldn’t think of how to dye the molecules into different colors so I just published the idea and forgot about it. Then I went to work for my dad’s machine tool company, but after 6 years I missed doing research so much that I decided to return to science.

**Figure:**
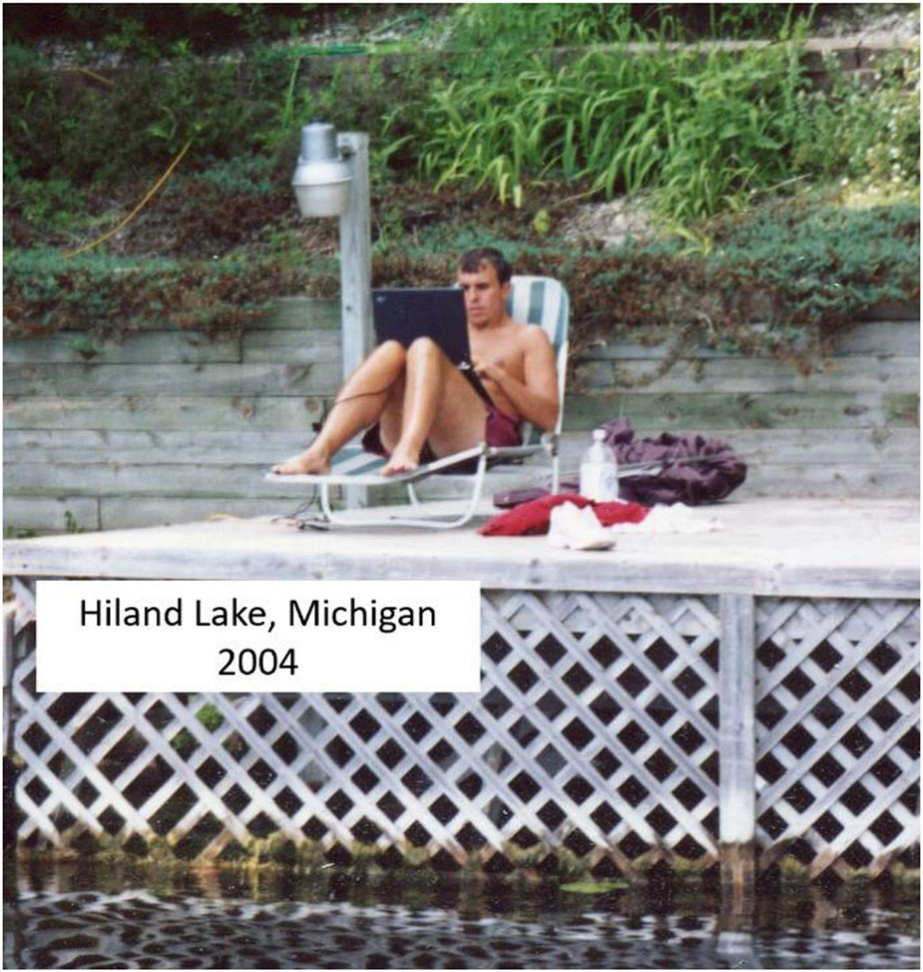
Eric, unemployed at his lakefront cottage and trying to relearn science after a gap of 10 years

I started reading scientific literature and came across a paper by Martin Chalfie on something called green fluorescent protein (GFP). The paper discussed about hijacking the DNA from a glowing jellyfish and splicing it on to any protein you want to see, and also hijacking the cellular machinery to produce copies of that protein with a glowing tag already on it, which was both revolutionary and elegant. They completely revolutionized cell biology, and got the Nobel in 2008, very deservedly. Based on that technology, I came up with an idea about using plane wave illumination from discrete directions to create a massively parallel array of foci which I called optical lattice microscopy, and contacted my best friend from Bell Labs Harald Hess to see if he could help me find a lab where I could work. Together we visited Mike Davidson at Florida State University where he had built one of the largest libraries of different fluorescent proteins, and he told us about photo activated green fluorescent protein (PAGFP), fluorescent protein that can be turned on and off with light. Harald and I realized that this protein would work with my idea of achieving super-resolution by dying molecules different colors, but instead of colors just have fluorescence turned on at different times. So we pooled our money together and built a microscope in 2 months in Harald’s living room and tested it with Jennifer Lippincott-Schwartz and George Patterson at NIH who developed PAGFP. Then we submitted our paper to *Science* and the rest is history. So that’s how I got back into science. That’s how I got the Nobel Prize.

**Figure:**
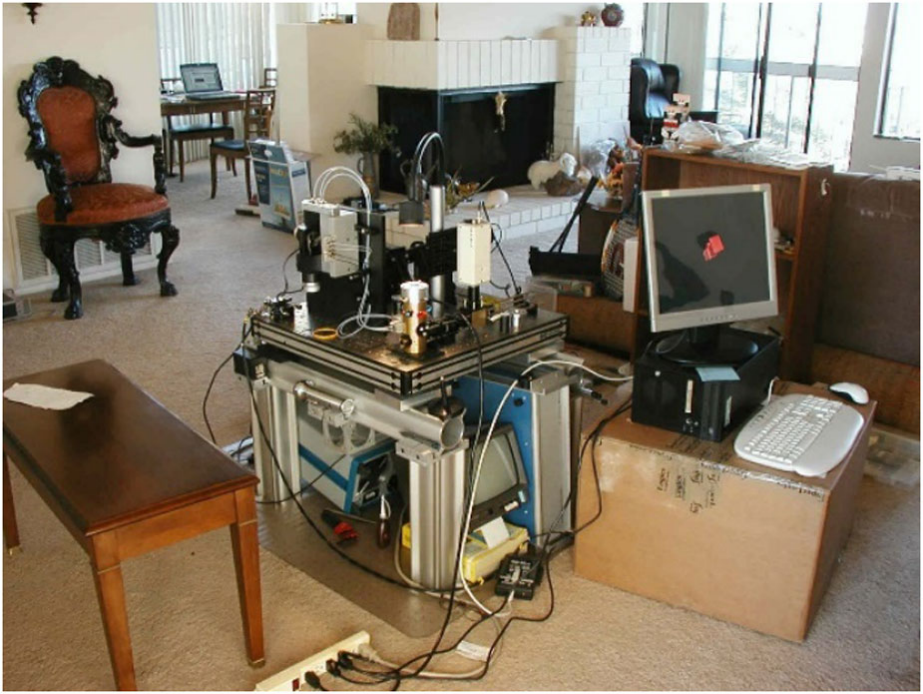
The original PALM microscope in Harald’s living room

**Figure:**
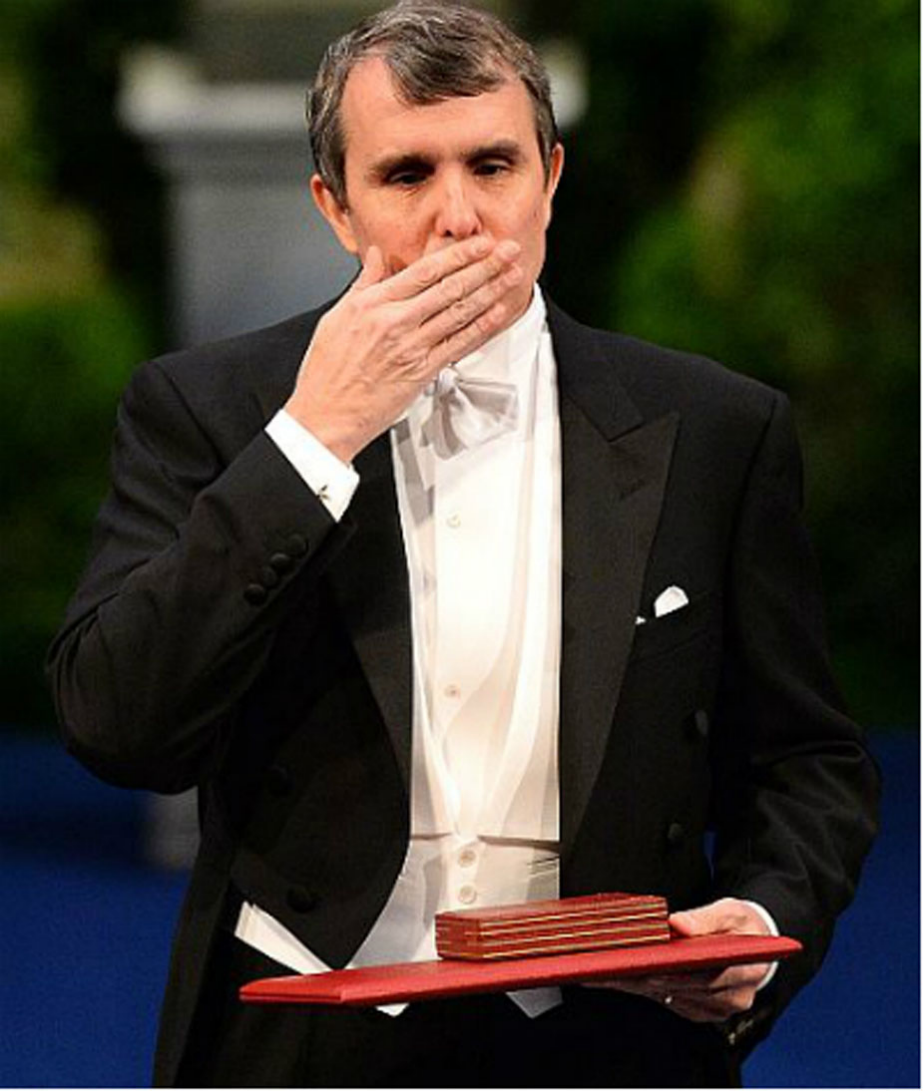
Eric, blowing a kiss to his wife from the stage in Stockholm with his Nobel medal and diploma in hand

**Figure:**
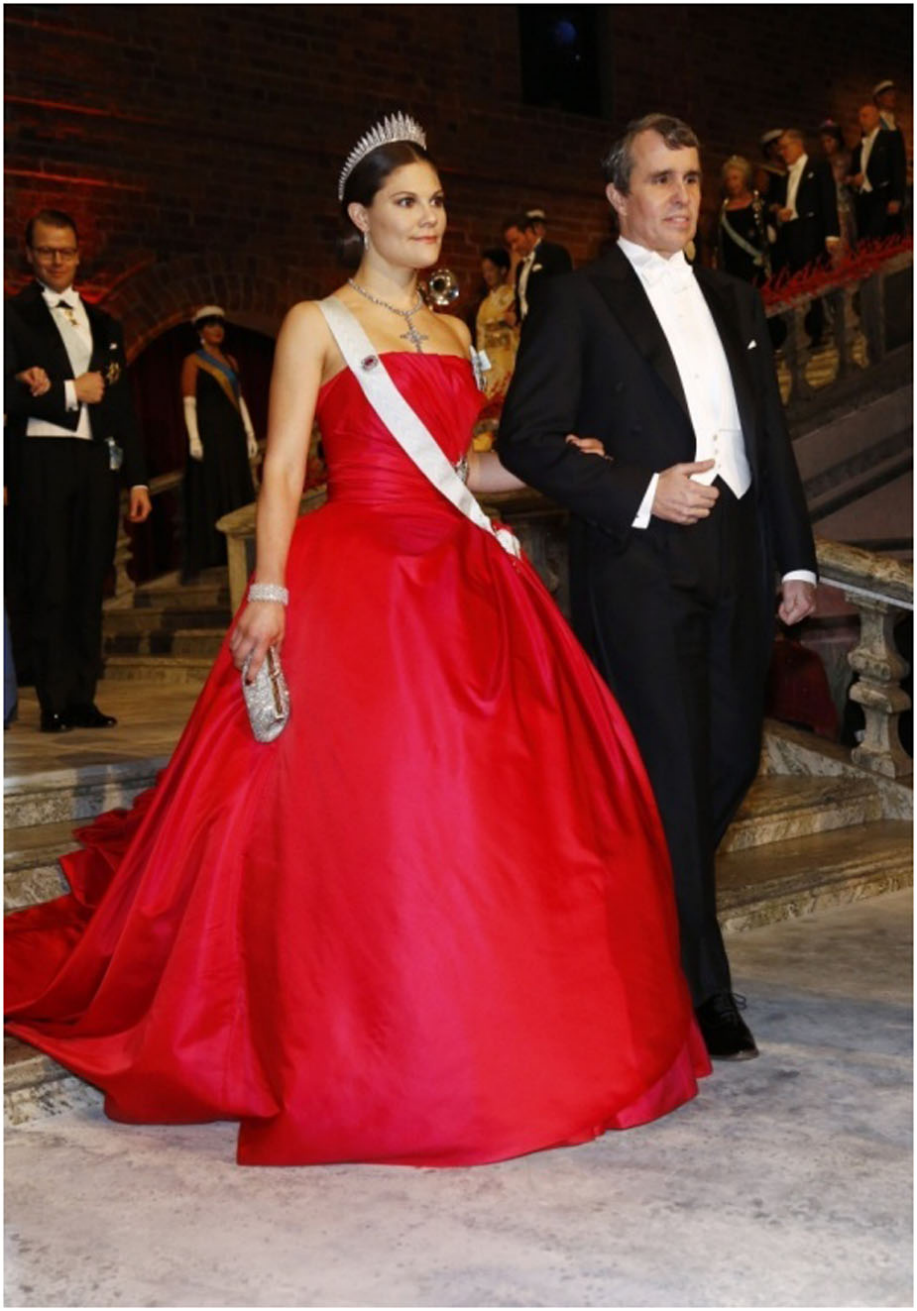
Escorting Crown Princess Victoria at the Nobel Banquet

**2. Could you talk about your experience working for your dad’s company?**

Prof. Betzig: I worked on several technologies in my dad’s machine tool company. One was using cameras to tell if a machine part was machined correctly. It was basically doing sub-pixel localization of features on a part using the same principles of sub-pixel localization in PALM, but now for figuring out whether the holes and surfaces on a machine part are in the right place or not. This is how you can borrow from one completely different discipline for another discipline. Then I came up with an idea on how to make a super productive machine that would manufacture auto parts with some pretty novel principles to it. I spent years developing that, but when it went to the marketplace, it was a complete disaster. Nobody wanted to buy it because it was too weird and too different. It was a risk for them, and that’s a very risk adverse business.

Which is why I’m astounded with the success of Elon Musk with Tesla, because the car business was as conservative as it can be. And the fact that he basically has dragged them kicking and screaming to electric vehicles is astonishing. That’s not even getting into his accomplishments with SpaceX, which are even more astonishing because that business was also very conservative and he reinvented it. But I’m not Elon Musk and I failed in my attempt to do that. So in 2002, I quit my dad’s company.

**Figure:**
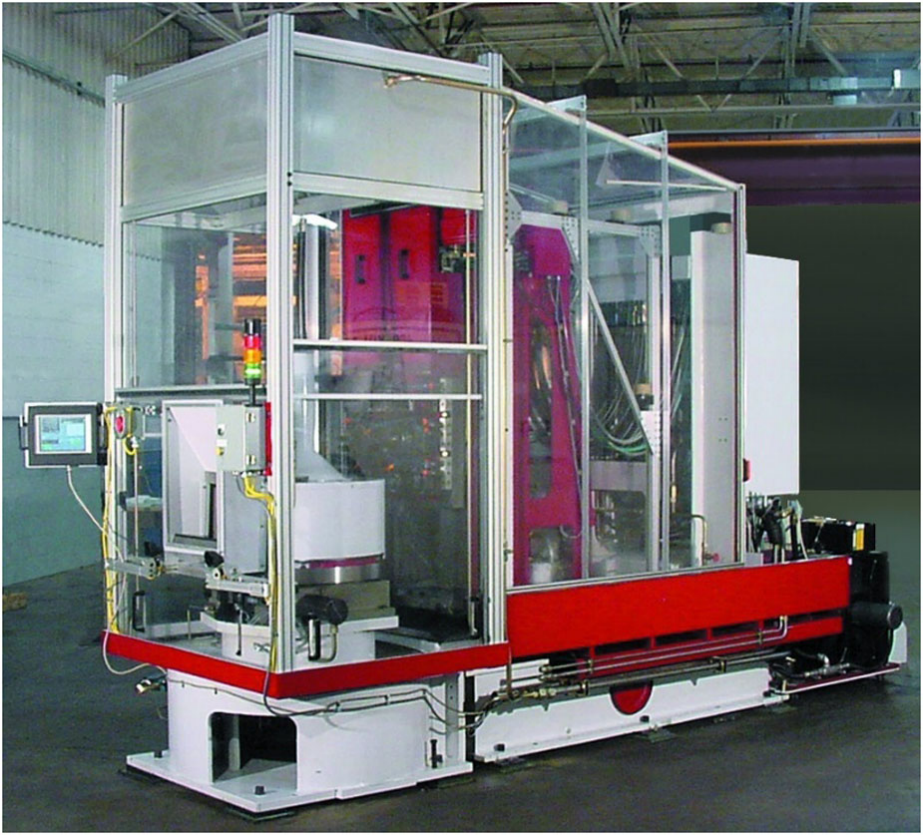
Flexible Adaptive Servohydraulic Technology (FAST) machining center Eric developed in his father’s company

**3. You were trained as an optical scientist, are there people who wonder why you were awarded the Nobel Prize for Chemistry?**

Prof. Betzig: That’s right. And I don’t know any chemistry, haven’t done chemistry since high school. I think in science you need to work out of your comfort zone. I’m not a molecular biologist, nor a chemist, but by working out of your comfort zone and having antenna up for new ideas, you could see how things go. A good scientist always has a number of problems half-baked in his or her head at any time. I had the bones of an idea of PALM in 1994, and when I learned about PAGFP it was like the key going into the lock and unlocking it. You have to fill your mind with ideas and thoughts, and creatively think about how different things can be combined. I think that’s why I enjoyed working at Bell Labs so much, because it was a place where cell biologists, neurobiologists, computer scientists, material scientists all worked under the same roof and it was amazing what breakthroughs were made just having people talking to each other. So I want to warn against siloization in scientific research. Don’t just have people studying the same discipline in one institution, or don’t just have people chasing preset goals. Encourage outside thinkers. It’s good to have a goal, but you never know what’s gonna help you get to that goal. And sometimes goals may not have been intelligently chosen, and it can put blinders on you. You have to be flexibly minded in science.

**4. So being flexible minded is a quality a good scientific researcher should have, what other qualities are needed?**

Prof. Betzig: I believe to make a good scientific investigator, you need to be open-minded, and have the willingness and ability to talk to many other people of different disciplines, which is the secret to creativity and innovativeness. Knowing the problem is often half of the solution, so you should always have in your mind a set of important and impactful problems that you don’t know how to answer. Then, by putting yourself in a position where you are exposed to different people from different disciplines, you’ll find that creativity will come naturally because if you have the problem in your head, your subconscious starts to think about what you can do to solve this problem. I’m a firm believer that creativity does not happen in the conscious mind. It happens in the subconscious mind. You have to starve your subconscious. Pick a problem, focus on it every minute of the day, starve your subconscious of anything except that problem, so it has to focus on the problem, then an answer will come to you. That was how I came up with the idea of PALM in 1994. I was pushing my baby in the stroller, I wasn’t thinking about super resolution, but then the idea popped into my head. It came out of something deeper.

Louis Pasteur, the French microbiologist, once said, “Chance favors the prepared mind.” To succeed, you just have to work really hard, make sure your brain is completely immersed in the problem or a number of problems, and then luck will come. But luck does not determine whether you’ll be successful. Hard work determines whether you’re going to be successful or not. Luck determines what you will be successful at. So another secret of success is hard work, and this is something that my Chinese postdocs never had a problem with, but it’s a big problem in the US. It’s the only correlate I know to success. It has nothing to do with intelligence or where you’re working. It’s nonlinear, because the more you know, the more efficient you get in finding out even more. So it goes in pretty much an exponential curve, not a linear curve, with output.

**5. I am glad to hear your Chinese postdocs are all hardworking. In China we value hard work and often tell young people to persevere, but you seem to have benefited from walking away from a problem when you hit a wall because you returned to big success. Would you like to share your ideas?**

Prof. Betzig: Perseverance doesn’t mean staying in the same job forever, nor does it mean focusing on the same problem, because sometimes problems have dead ends. Perseverance means focusing on the science or what motivates you in your career. I will agree that the hardest thing for me has been to know when to quit for exactly that reason because you’re taught: persevere, persevere, persevere. So it was a tough decision to quit Bell Labs and an even tougher decision to leave my dad’s company. At Bell Labs at least I knew that I had taken that near field technique as far as it could go. I knew that I wasn’t going to be able to break the laws of physics. So it wasn’t as difficult a decision to say, “Forget perseverance, I’m quitting there.” Because I felt like I had done everything I could do.

With my dad’s company it was really hard because not only was it hurting my relationship with my dad, but I also just don’t know if things would work out if I had stayed 2 more years. I keep saying there’s an alternate universe somewhere in which I am an unemployed mechanical engineer living in Michigan, and it’s only by extreme luck that I got to where I am today. When I ended my Nobel talk, I dedicated it to all the people who took a risk and failed, because though you don’t hear about them, they are more important because there’re more of them than people who took a risk and succeeded. You can go a whole career having a bunch of good half-baked ideas and never find the key. But I still encourage my students to take risks. When you take a risk, particularly a risk for your career, it usually brings out your best self. When you take a risk and initially fail, you’ll work your absolute hardest to try to succeed and that’s when you find your limits. It’s being alive, about being truly alive as opposed to kind of just passing through life.

**6. So back in the 1980s, why did you choose microscopy as your subject of research in graduate school?**

Prof. Betzig: I felt I was very lucky to go to graduate school in 1982 and pick microscopy as a field. That was a lucky decision because it was right at the time that personal computers were available to automate microscopes, when the CCD chip was just becoming available, when fluorescence was starting to take off, and when lasers were cheap enough that they could go in individual labs. All the microscopes that have come out since 1980, whether its wide field, confocal, two photon, light sheets, super resolution, they are all about taking all of those things that appeared around 1980 and just stirring them in different combinations to get new microscopes. So being at the start of your career in 1980 and choosing microscopy is like being a physicist in 1920 and deciding to study quantum mechanics. That’s the way microscopy was in 1980, just a fruit waiting to be plucked. I was just lucky to have picked the right area.

**7. Was it your dream to engage in optical research since childhood?**

Prof. Betzig: I wanted to be an astronaut ever since I was a kid because I grew up with the Apollo program and the flights to the moon then. I went to California Institute of Technology (Caltech) specifically to get enough training to be a scientist astronaut. But by the time I graduated in 1982, the space shuttle was flying and I knew the space shuttle was a really bad idea. It was clear that this was a dead end but it was politically driven to make this monstrosity that was the space shuttle, and I was proven right in the end. So I had to pick something different and I always wanted to do something that I thought would be big, not incremental. And so when I went to Cornell University and my advisors wanted to do this super resolution with near field, it had a potential to be revolutionary and super resolution eventually got a Nobel Prize, so that intuition was right. It was not the right technique then, but it was the right idea. I wanted to be an explorer, I wanted to be on the Moon and Mars, but if I can’t go outward, I can explore inward. I would say one of the most gratifying things about being in microscopy is the biology - being able to reveal biology and being able to see things. We’ve worked with 100 different groups and everyone comes with some specimen that they’ve been studying for their entire career, and then they see it in our microscope and they’ve seen it in a way they’ve never seen before. And it excites them and it excites me. Nature is really beautiful, extremely beautiful, extremely complex. I’ve had many points in my career where I have felt like Galileo, who sees the phases of Venus, he sees the Moon isn’t a sphere but has mountains. Everywhere he pointed that thing, he made a discovery. With our microscopes, there have been times in which every specimen we put in revealed new biology and beautiful biology.

**8. In 2019, you and your colleagues created a “Swiss Army Knife” mega-microscope. Could you give us an update on its progress so far?**

Prof. Betzig: The Swiss Army Knife, which we call MOSAIC, is a successor to the lattice light sheet with adaptive optics (lattice AO). For years, a big focus of my lab has been to get the technology into the hands of biologists. Most microscopes never leave the optics person’s bench, and it’s useless if it just sits on your bench and not on the bench of biologists. We’ve taken many steps to change that. With the lattice light sheet microscope, we first created a version that others could replicate. About 130 different groups so far have asked for and received information on how to do that. I would guess of those 130, about 30–40 have actually built them. And of those 30–40, I would say probably 15–20 are actually in fairly common use. A lot of the microscopes will be used for a little while and then not used. So we want to get enough people to use it, get enough applications done and enough biological findings created so that a commercial company would want to build it. They will make the microscopes turnkey, which means you just put the sample in and press a button and you get your data. Also, when a commercial microscope breaks or if the biologists need advice on how best to use it, there’s somebody to call in the company who can help. That’s the only way biologists will use microscopes routinely.

The original lattice AO microscope cost us over $1 million to build and covered a ten foot optical table, something that could never be a commercial success. So we wanted to make a next generation version that is cheaper to make and easier to use. We eventually realized that when we had paid the price for all the components needed for lattice AO, we also had all the components needed to do nearly any form of modern optical microscopy, but with adaptive optics too. Therefore, why not make it a microscope that can do everything? Because if you go to biology centers, you will see that though they have many different microscopes, most are not used. So we built one microscope that can meet all the biologists’ needs, it can change from a confocal to a two photon or a light sheet at the press of a button. Tian-Ming Fu, the postdoc who put it together, is now at Princeton. We still haven’t published the paper, because he has to find the time to write the paper up and then we can put it to bed and be done with it. In the meantime, though, we’ve given the plans to nearly 40 different groups, and a number of them are in the process of assembling and using it.

**9. A lot of the instruments in laboratories are so complicated that postdocs had to go through a lot of training in order to operate them well, but as you mentioned, if you can have customer friendly and easy to operate instruments, that will be very important.**

Prof. Betzig: Yes, it’s critical for biology. You can’t meet biologists halfway, you have to go all the way to give the biologists what they want. You can’t get them to come out of their comfort zone, because that just won’t work. And to make that happen, again you have to be informed. They don’t know what’s technically possible, but they know what they want scientifically. That means that you have to talk to them about their wants, but you also have to think about the things which they don’t know that they want but they would want if they know what’s possible. So you have to have a dialogue with them to say if I could deliver this, would that be of interest and then decide from that basis whether you want to go and make the widget you want. Don’t just make the widget because you want to make it or you want to publish a paper. That’s not very valuable.

**Figure:**
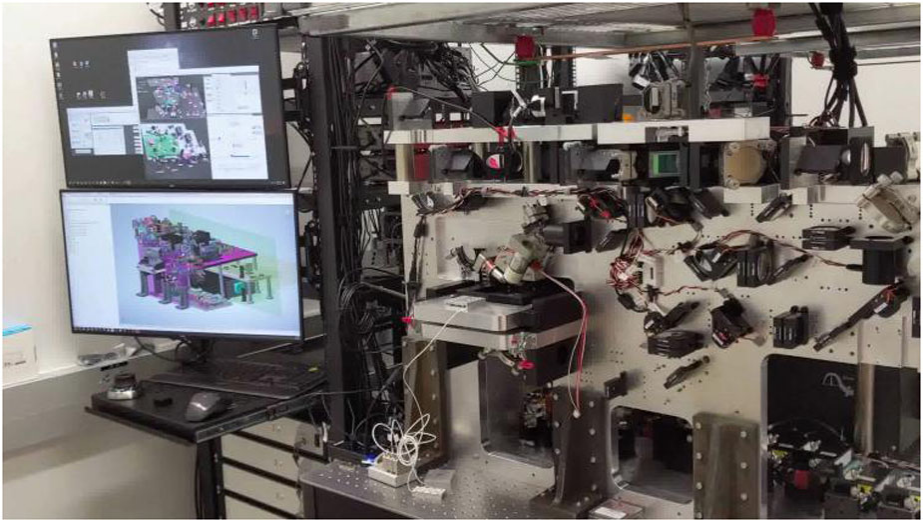
The MOSAIC “Swiss Army Knife” microscope

**Figure:**
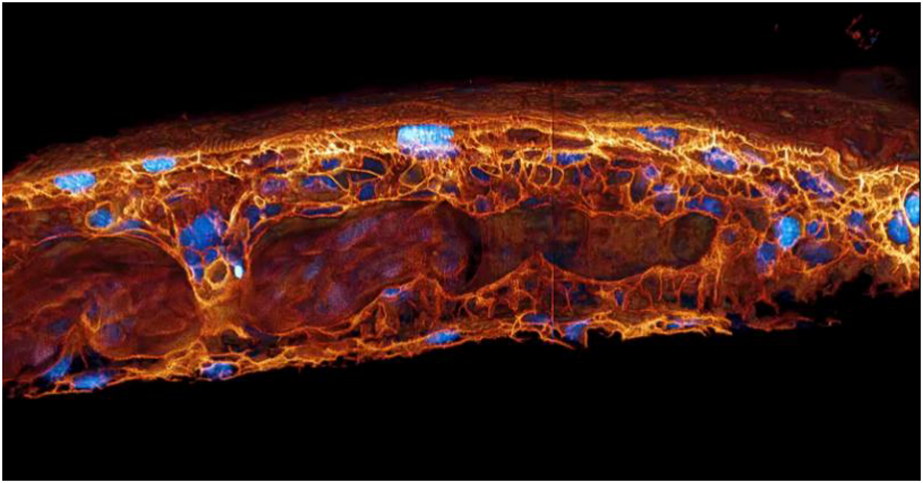
MOSAIC 3D cutaway view of blood flow in the tail fin of a developing zebrafish

**10. In 2021, you co-founded Eikon Therapeutics. How do you think scientific research and achievement transformation should be linked?**

Prof. Betzig: Eikon Therapeutics came out of an experiment we did with the lattice light sheet with biologist Robert Tjian who’s also at Berkeley. He is a biochemist, and in biochemistry they had figured out all the various proteins that have to come together to start the process of transcription, that is how a polymerase is recruited to the start of a gene on DNA and then spit out RNA, and they wanted to observe this process. We realized when we used PALM to track the kinetics of transcription factor molecules that all the necessary the molecules don’t all come together at the same time. In fact, there are many copies of every type of molecule, which will bind to the DNA just for a split second and then go away and then another one comes. Nothing like the semi-static process the biochemists imagined. Instead, the molecules all buzz around under Brownian motion and bind for a second or so and then the next one comes and the next one until the polymerase is eventually recruited. In my opinion this may be the single biggest scientific finding of super resolution so far. It upturned the mental picture of how transcription works, and transcription is important because many diseases are caused by transcriptional errors. Tiny changes to the protein could have big impacts. Tjian thought the technology would be great for drug screening, putting in different compounds and watch how key proteins change their kinetics under different drugs that you apply. So we started a company, Eikon Therapeutics, and were able to recruit a bunch of extremely high profile, high powered people in the drug business. I don’t have any day-to-day role. I helped a little bit to get the screening going because I know how to build microscopes, but the big part of the business is way above my knowledge level. It’s in good hands, but there’s no guarantee. Every startup is extremely high risk, most end up nowhere in the end. We’ll see where it goes. But it was an interesting experience to learn how the whole Silicon Valley venture capital world works. It was very different from my normal experience, so that was educational to say the least.

**Figure:**
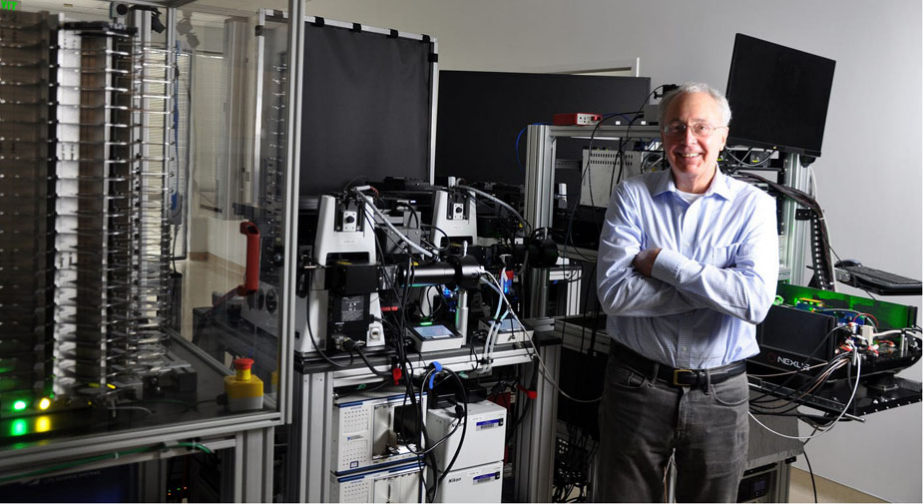
Roger Perlmutter, CEO of Eikon Therapeutics, with a portion of Eikon’s high throughput single molecule drug screening pipeline

**11. Who would be your science heroes?**

Prof. Betzig: From a technical business sense, I think Elon Musk is my hero. I’ve never seen a guy who takes on more big risks than him. He’s very inspirational, just an incredible risk taker. The last person I know who was even remotely like that was Howard Hughes, so he is the 21^st^ century version of Howard Hughes.

From a science side, I don’t really have a hero. One of the sobering things I feel about winning the Nobel Prize is I’ve had close contact with lots of famous people, scientists, politicians, etc. It’s scary how ordinary most of them seem. They’re not smarter. They’re not more beneficent. They don’t have deeper thoughts. They’re just people. People are people and whether they’re Nobel laureates, the President of the United States, or a billionaire, they’re all people and humans are very limited. Often, when people have success, they tend to forget their limitations, and extrapolate from their success in one area into expertise or proficiency in other areas in which they are not expert. I’m very cognizant of my many limitations, and the only time I’m willing to talk authoritatively is about microscopy because that’s the one area I feel like I’m competent to have an opinion on.

**12. Do you ask anyone for advice before making big decisions or do you just trust your own instinct?**

Prof. Betzig: I will certainly ask for advice, both advice on a small level about details and advice on a big level. A lot of my success in my career has been because of my friend Harald. Harald and I met on the first day I interviewed at Bell Labs in 1988 and we immediately became best friends. We worked 14 hours a day every day. We played tennis every day. We ate dinner together every day. We talked science constantly all that time. Then I left and then he left and I was in Michigan and he’s in San Diego, we kind of fell out of contact. But when I was trying to get back into science, the first guy I contacted was Harald because I needed a sounding board and I needed advice on how to get back in. And that led to the trip to Florida State, which led to us working together again. We both ended up at Janelia for the whole time I was at Janelia. We continued to hang out together, and had a paper in 2020 in *Science* on a cryogenic correlative super resolution and 3D electron microscopy. At many points of my career, I would have been sunk without Harald as my friend and mentor. He’s still my best friend, and the most talented physicist I’ve ever known.

**Figure:**
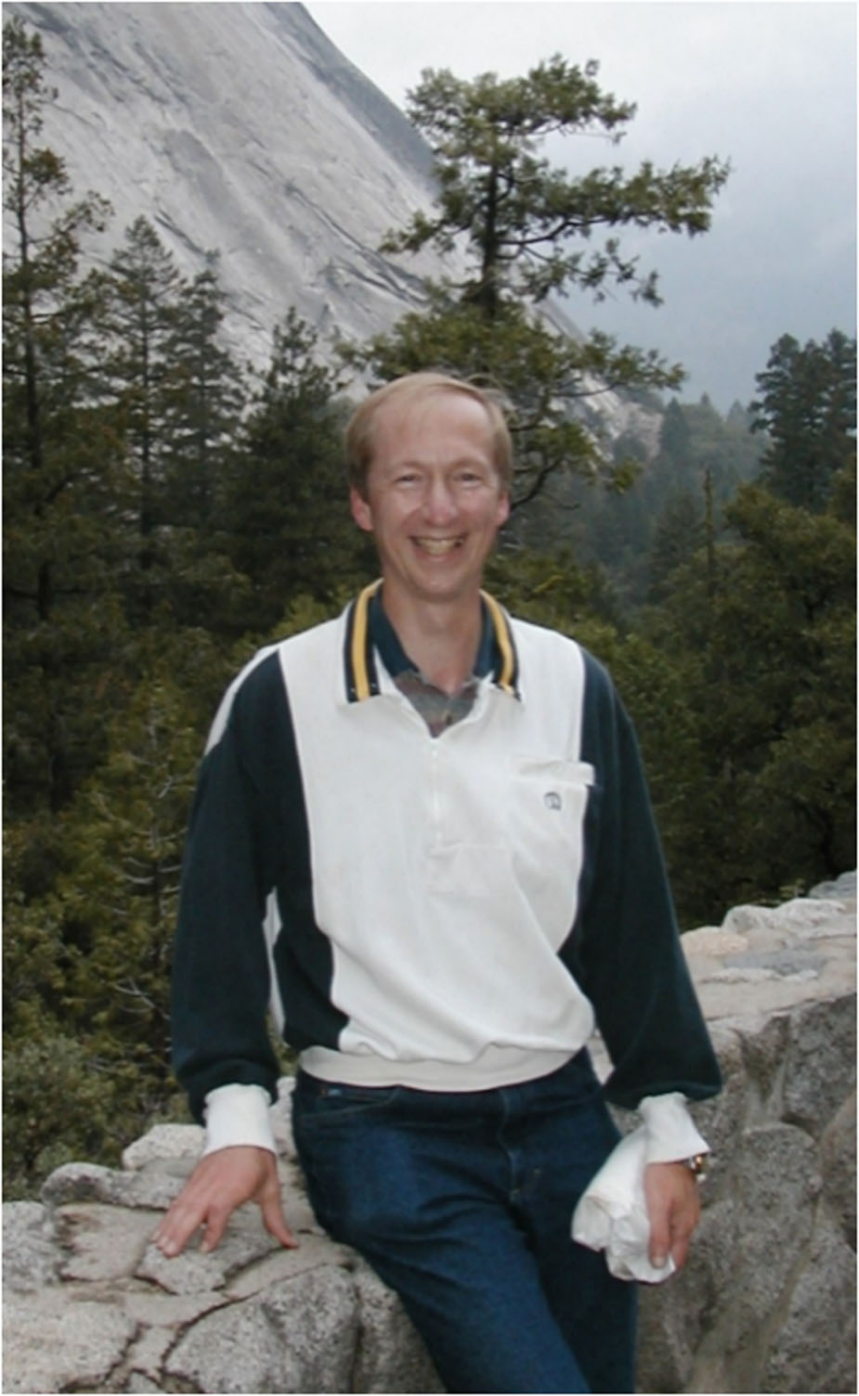
Harald, during one of their brainstorming hikes in Yosemite National Park

**Figure:**
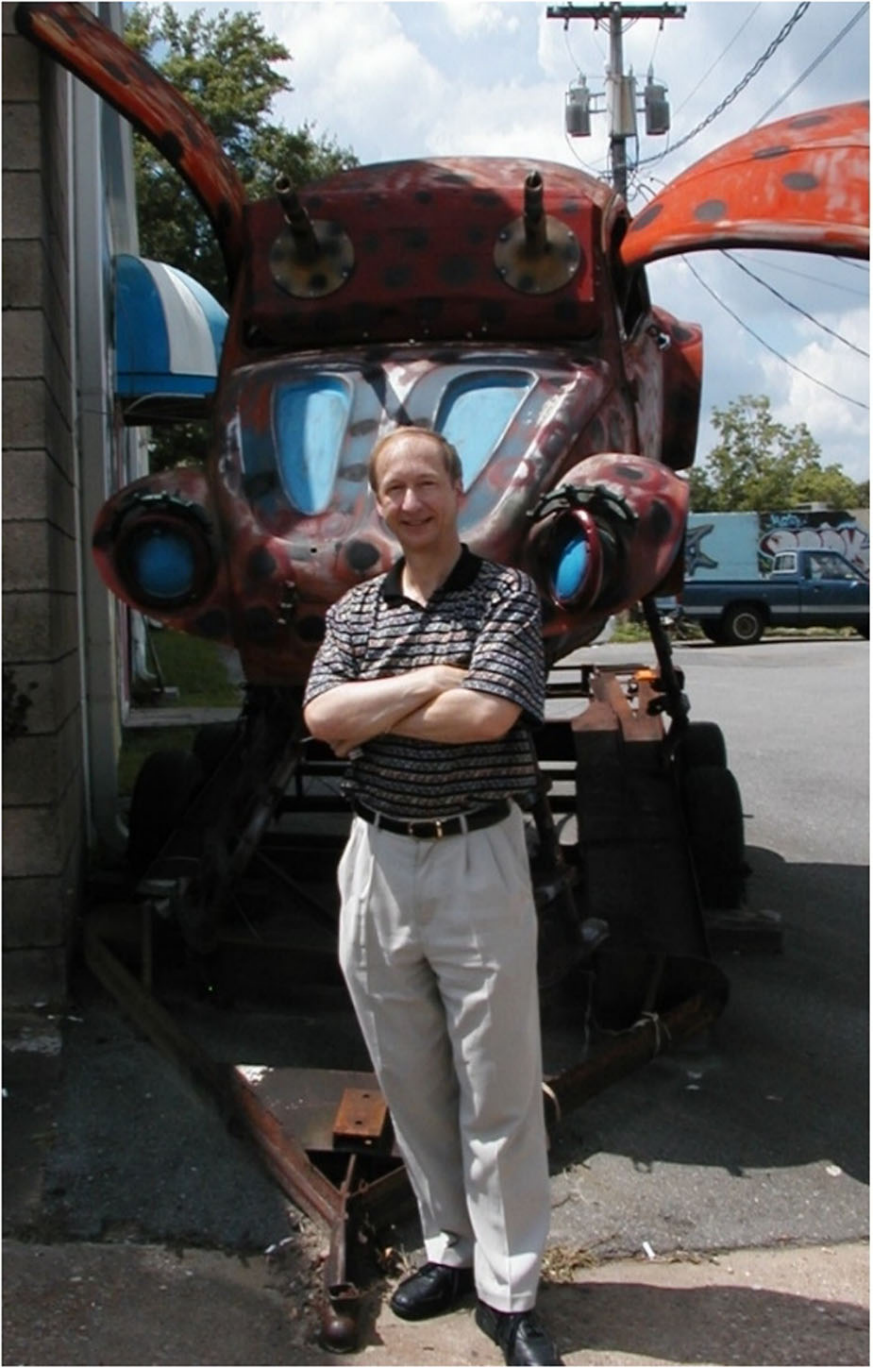
Harald, in Tallahassee, FL on the trip where they conceived the idea for PALM

**13. Since we are talking about publishing papers, it will also be our honor if you could submit your paper to** ***Light: Science & Applications*?**

Prof. Betzig: Well, you know the way it is with postdocs, they want *Science*, *Nature* or *Cell*. So that is where we generally try first, if it’s a big new microscope. This is totally stupid. But the reasons we published a lot in *Science* is because every *Science* paper gets another postdoc the job he wants, and it draws the attention of many biologists who read *Science* and might be able to make good use of the technique. It’s that simple. I don’t think it’s good, but it’s the way of the world and I owe my postdocs for the 5 or 6 years that they sweat to get the results. *Light: Science & Applications* is affiliated with *Nature*, the affiliation is helpful, and you guys have come a long way very quickly.

**Figure:**
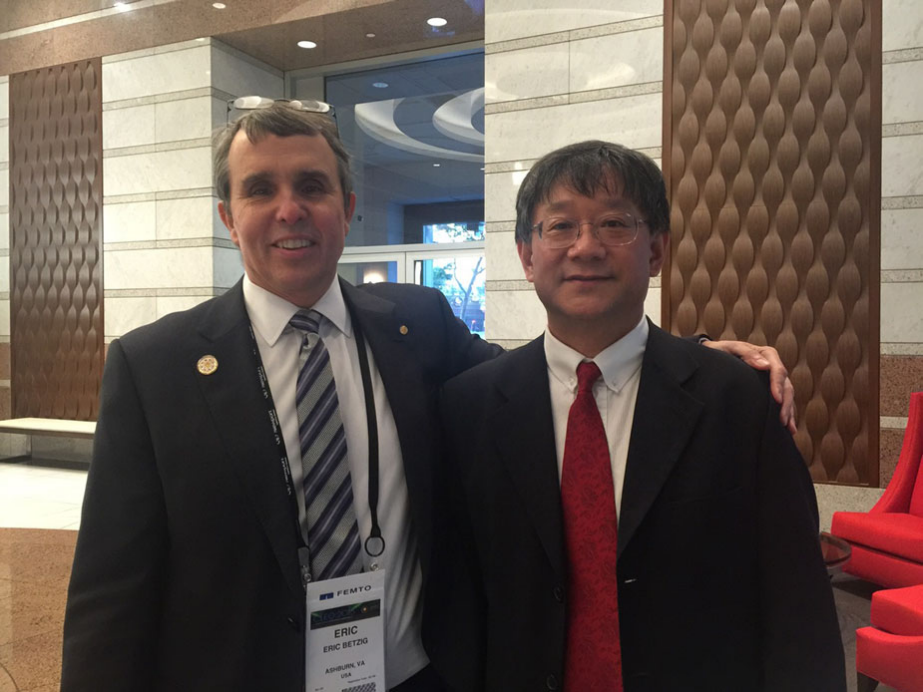
Eric and Prof. Xi-Cheng Zhang, Co-Editor-in-Chief of *Light: Science & Applications*

**14. What do you think of Chinese students?**

Prof. Betzig: Most of my postdocs at Janelia have been from China and they have been great. I’ve been blessed with really great postdocs. But I can already see that China doesn’t yet seem to reward risk-takers, but rather those who publish lots of papers in journals with high impact factors. For example, if you’re in an institute of optics, you hang out with a lot of optics people. How much do you hang out with chemists and biologists and data scientists and mathematicians and so forth? That’s why I always loved Bell Labs, where cell biologists, neurobiologists, computer scientists, material scientists all worked under the same roof and ate lunch together at the same table.

**15. When you face difficulties in work and life, how do you adjust yourself mentally?**

Prof. Betzig: That’s a good question. I always have difficulties. I rely on my wife for things related to life, and I rely on Harald for things related to science and that works pretty well.

**16. What are your hobbies?**

Prof. Betzig: I have five kids, three of whom are still young. Most of my hobbies are just taking care of the kids and taking them to swimming lessons, tennis lessons, school, all that stuff. That and work, and occasionally hiking and tennis when I can. I always say that guilt rules my life because when I’m working, I feel guilty I’m not with the family and when I’m with the family I feel guilty that I’m not working. So there is not a hell of a lot of room left for anything else because if I do anything else like hiking, then I feel doubly guilty because I’m not at work or with the family.

**17. What advice and suggestions would you like to give to young researchers?**

Prof. Betzig: Don’t be afraid to take risks. There’s always a way to recover from a failure. Life is short, so live it with all the vigor and creativity you can.

